# An integrative study on the impact of highly differentially methylated genes on expression and cancer etiology

**DOI:** 10.1371/journal.pone.0171694

**Published:** 2017-02-08

**Authors:** Bugra Ozer, Ugur Sezerman

**Affiliations:** 1 Biological Sciences and Bioengineering Program, Faculty of Engineering and Natural Sciences, Sabanci University, Istanbul, Turkey; 2 Department of Biostatistics and Medical Informatics, Acibadem University, Istanbul, Turkey; University of North Carolina at Chapel Hill School of Medicine, UNITED STATES

## Abstract

DNA methylation is an important epigenetic phenomenon that plays a key role in the regulation of expression. Most of the studies on the topic of methylation’s role in cancer mechanisms include analyses based on differential methylation, with the integration of expression information as supporting evidence. In the present study, we sought to identify methylation-driven patterns by also integrating protein-protein interaction information. We performed integrative analyses of DNA methylation, expression, SNP and copy number data on paired samples from six different cancer types. As a result, we found that genes that show a methylation change larger than 32.2% may influence cancer-related genes via fewer interaction steps and with much higher percentages compared with genes showing a methylation change less than 32.2%. Additionally, we investigated whether there were shared cancer mechanisms among different cancer types. Specifically, five cancer types shared a change in AGTR1 and IGF1 genes, which implies that there may be similar underlying disease mechanisms among these cancers. Additionally, when the focus was placed on distinctly altered genes within each cancer type, we identified various cancer-specific genes that are also supported in the literature and may play crucial roles as therapeutic targets. Overall, our novel graph-based approach for identifying methylation-driven patterns will improve our understanding of the effects of methylation on cancer progression and lead to improved knowledge of cancer etiology.

## Introduction

Recent advancements in omics technologies have enabled increasing numbers of studies to explore the roles of epigenetic factors in the mechanisms of different diseases, including cancer [1–3]. One of the epigenetic mechanisms involved in disease etiology is alteration of DNA methylation levels, which has been proven to play a vital role in gene expression regulation. However, the interplay between methylation and expression and how this interplay can be tied to cancer-driving mechanisms in various types of cancers has yet to be thoroughly investigated.

The general idea about the effect of methylation on expression is that methylation plays crucial roles in gene silencing and the regulation of gene expression. It is well established that hypermethylation in promoter regions leads to inactivation, whereas hypomethylation is associated with genomic instability and loss of imprinting, in addition to contributing to cell transformation and the progression of lesions, which may be the key factors in the reproduction and metastasis of cancer cells [4]. Indeed, both hyper- and hypomethylation have previously been associated with a variety of cancers, including kidney, colon, pancreas, liver and lung cancers [5–15]. Although the opposite pattern has also been observed in several studies [16, 17], an inverse correlation is expected between changes in methylation levels and expression [18–20]. A recent study by Lee et al. demonstrated that there is a tendency toward direct correlations in backbone regions, whereas inverse correlations are expected near CpG sites in promoter regions [21]. Additionally, gene silencing via the hypermethylation of tumor-suppressing genes and activation of tumor-promoting genes via hypomethylation has been demonstrated to favor oncogenesis [22]. Because DNA methylation is reversible (unlike genomic alterations), these genes may represent promising candidates for new therapeutic strategies [8, 23].

In methylation-based studies, after differentially methylated genes are identified, the next step is the determination of how these genes are involved cancer development mechanisms. To this end, it is essential to identify the pathways that are affected by these methylation-driven changes. In a recent study, Gevaert et al. developed a univariate beta mixture model-based method for the identification of differential methylation, termed MethylMix [24], to explore transcriptionally predictive methylation-driven genes and pathways in twelve different cancers. Alternatively, Kim et al. proposed a logistic regression-based method for gene set enrichment, termed LRpath [25], for the investigation of important methylation-driven pathways. Moreover, pathway similarities across different types of cancers are included in this analysis.

In numerous studies, transcriptomics experiments have also been incorporated into DNA methylation experiments so that the correlations, in addition to the differences between methylation and expression can be used to obtain information about disease-causing mechanisms. However, when incorporating expression information into methylation studies, the use of matched tissue samples alone is not sufficient; instead, it is crucial to use paired tumor and control samples from same patient. Because only tumor or control samples may exhibit distinct expression patterns, the use of paired data and the performance of methylation and expression analyses on these data will improve the avoidance of both false-positive and false-negative results [26].

To study the role of methylation in cancer development mechanisms, one must explore how oncogenes and tumor suppressor genes (drivers) are modified or how differentially methylated genes alter the expression levels of driver genes through a set of interactions in a protein-protein interaction (PPI) network. In the present study, we defined a novel graph-based analysis strategy for identifying methylation-driven potential cancer-causing gene patterns. In total, we applied our method to six different cancer types: cholangiocarcinoma (CHOL), colon adenocarcinoma (COAD), kidney renal papillary cell carcinoma (KIRP), liver hepatocellular carcinoma (LISC), lung squamous cell carcinoma (LUSC) and thyroid cancer (THCA), using the Illumina HumanMethylation450k methylation chip and RNA sequencing data. In contrast to previous methods, to avoid false-positive and false-negative results, we included only paired samples in our analysis. To extract the significantly altered methylation-driven patterns within a STRING protein-protein interaction network, we first defined a methylation change threshold of 32.2% for “large methylation changes”. Subsequently, in addition to focusing on the interplay between methylation and expression, we carefully considered the individual relationships between different genes to ensure a deeper understanding of the methylome and transcriptome. Overall, our study not only defined a novel approach for the identification of significantly altered methylation-driven pathways but also contributed to improving our knowledge of the etiologies of different cancers and the common and distinct features among them.

## Materials & methods

### Dataset

In this study, we conducted analyses of methylation and expression data from six different cancer types retrieved from the Cancer Genome Atlas (TCGA), which included thyroid cancer [27], lung squamous cell carcinoma [28], kidney renal papillary cell carcinoma [29], colon adenocarcinoma [30], cholangiocarcinoma (provisional), and liver hepatocellular carcinoma (provisional). While treating each cancer type individually, we selected only samples that were matched to the anatomic site of the tumor. Moreover, because our approach is sensitive to possible noise factors due to the integration of methylation and expression information, we only included samples with available data on both control and tumor samples from both the Illumina HumanMethylation450k Chip and RNA sequencing (RNA-Seq). We included a total of 92 THCA samples, 18 CHOL samples, 30 COAD samples, 46 KIRP samples, 14 LUSC samples and 78 LIHC samples in the datasets. The methylation data consisted of intensity values matching CpG sites covering different regions of the gene, whereas the RNA-Seq data consisted of count values corresponding to each gene that were computed by the data owners.

### Methylation analysis

Methylation is a region-specific, rather than a gene-specific phenomenon; hence, methylation in different gene regions can lead to diverse consequences. In our methylation analysis, we benefited from the ChAMP pipeline [31], which included in the R-Bioconductor package and is specifically designed for the analysis of Illumina HumanMethylation450k chip data. ChAMP employs a sliding window approach (Probe Lasso) for annotating CpG regions with genomic locations [32]. CHAMP allows of the investigation of methylation occurring in different genomic regions, including in the first exon, 3'UTR, 5'UTR, gene body, intergenic region and within 200 bp and 1500 bp proximities of the transcription start sites. Moreover, the beta values associated with each methylation change are used as estimates of methylation levels, which is the ratio of the methylation probe intensity to the overall intensity and provides an intuitive biological interpretation. [21]

After downloading the methylation intensity data from TCGA, the BMIQ normalization method [33] was applied to avoid the bias introduced by the Infinium type 2 probe design. The magnitude of the batch effects was corrected using the ComBat normalization method, which is an empirical Bayes-based method of correcting for technical variation related to a slide [34]. Moreover, because possible polymorphisms in an individual’s genome may affect the methylation status of probes, we excluded SNPs with frequencies greater than 0.05 based on the 1000 Genomes Project [35].

After pre-processing, analyses of copy number aberrations (CNA) and the segmentation of methylation variable positions (MVPs) into biologically relevant differentially methylated regions (DMRs) were conducted using the “champ.MVP” function of the CHAMP package. When conducting the analysis of copy number aberrations, we focused on the entire gene, rather than only including particular genomic regions. Individual tumor samples were evaluated against pooled normal samples, and the corresponding regions and segmental mean changes were reported in the output of the analysis. To determine whether copy number aberrations led to corresponding expression changes for the same gene, we used a segmental mean change of 2 as the threshold. Moreover, we annotated genes that exhibited both increases and decreases in different samples from the same dataset as “not important”.

In contrast, to avoid false-positive results in the differential methylation analyses, the Benjamini-Hochberg calculation [36] was applied for all p-values, and genes with detected Benjamini-Hochberg false discovery rates (FDR) below 0.1 were selected as “differentially methylated”.

Additionally, the Illumina HumanMethylation450k chip provides information about more than 450,000 different regions predicted in approximately 22,000 genes in the human body. Consequently, there is usually more than one differentially methylated region that falls within the borders of a given gene, which causes discrepancies in the data. To solve this problem, we evaluated regions of differing methylation within each gene and defined a “general trend of change” for each gene by checking whether the majority of changes were upregulation or downregulation. Depending on the direction of the change, the CpG region exhibiting the greatest methylation change, an FDR below 0.1, and a change in the same direction as the general trend was taken as representing the change in the methylation level for the whole gene.

To investigate the effects of large methylation changes, we first defined “large methylation change” threshold. For this purpose, we pooled all of the data and identified the distribution that best fit the data using the “fitdistr” function of the MASS R package [37]. Thus, the Cauchy distribution was found to best explain the data. We calculated the central value and scaling parameter for the pooled data as the Cauchy distribution parameters. For central value estimation, we took the truncated mean of the middle 24% of the sample order statistics, which has been demonstrated to be valid for the Cauchy distribution [38]. In our pooled data, we detected a central value of 16.8%. In contrast, the log-likelihood function was used for the scaling parameter. The corresponding log-likelihood formula can be found below:

$$\sum_{i=1}^{n}\frac{\gamma^{2}}{\gamma^{2}+{\left[\chi_{i}-\chi_{0}\right]}^{2}}-\frac{n}{2}=0$$

where n is the sample size; y is the scaling parameter; and *x*_0_ is the central value. After the calculation of each sample value, we identified a scaling parameter of 7.77. We used the central value plus two scaling parameters away from the center as the “large methylation change threshold”; thus, we set 32.2% as the high methylation threshold.

### Expression analysis

RNA sequencing analyses for all cancer types were performed using the edgeR [39] Bioconductor [40] package. The raw RNA sequencing reads associated with each sample were not available on the TCGA Server; hence, the quality control, pre-processing, mapping and counting procedures were performed by the providers of the data [27, 30, 41, 42]. We worked on counting the data produced via the RSEM procedure [43], and we applied EdgeR for the detection of differential expression between the tumor and control samples. EdgeR benefits from empirical Bayes estimation and tests based on the negative binomial distribution [39]. Similar to the methylation analysis, we performed Benjamini-Hochberg corrections [36] for all p-values. Finally, genes showing a fold-change >2 were selected as “differentially expressed” for our analysis.

### Suppressors and oncogenes

In our analyses, to investigate the potentially cancer-causing set of interactions, we searched for validated oncogenes and tumor suppressors in the literature. For this purpose, we benefited from 398 genes that were included in KEGG: Pathways in Cancer list [44]. Moreover, we added 5 genes to this list that were included in the QIAGEN Human Oncogenes and Tumor Suppressor Genes RT^2^ Profiler PCR arrays [45]. Overall, among the 403 available genes in the Pathways in Cancer dataset, 129 genes were annotated as either “tumor-suppressors” or “oncogenes” based on the Tumor Suppressor Gene Database (TSGene) [46] and the work of Vogelstein et al. [47]. Finally, further filtering was conducted because only 110 genes had available methylation values for all cancer types; thus, we used these 110 genes in the correlation analyses and the calculations of average distances from drivers.

### Integrating protein-protein interaction information

In this study, we extracted methylation-affected cancer-related patterns by examining protein-protein interactions close to the cancer-related genes. As there were not any well-defined directional, human protein-protein interaction network, we used the widely employed STRING database [48] because STRING covers approximately 9,000,000 proteins and provides information about the types of relationships that exist between pairs of proteins. As the type of interaction was crucial for our analysis, among the eight different interaction types that exist in the STRING database (i.e., activation, binding, expression, post-translational modification, inhibition, phenotype, catalysis and reaction), we considered only “activation” and “inhibition” relationships. Moreover, we filtered out non-human interactions and interactions with low and medium confidence (combined confidence scores<800). Ultimately, a total of 70,518 protein-protein interactions were included in further analyses.

### Driver distance calculation

**Fig 1:**
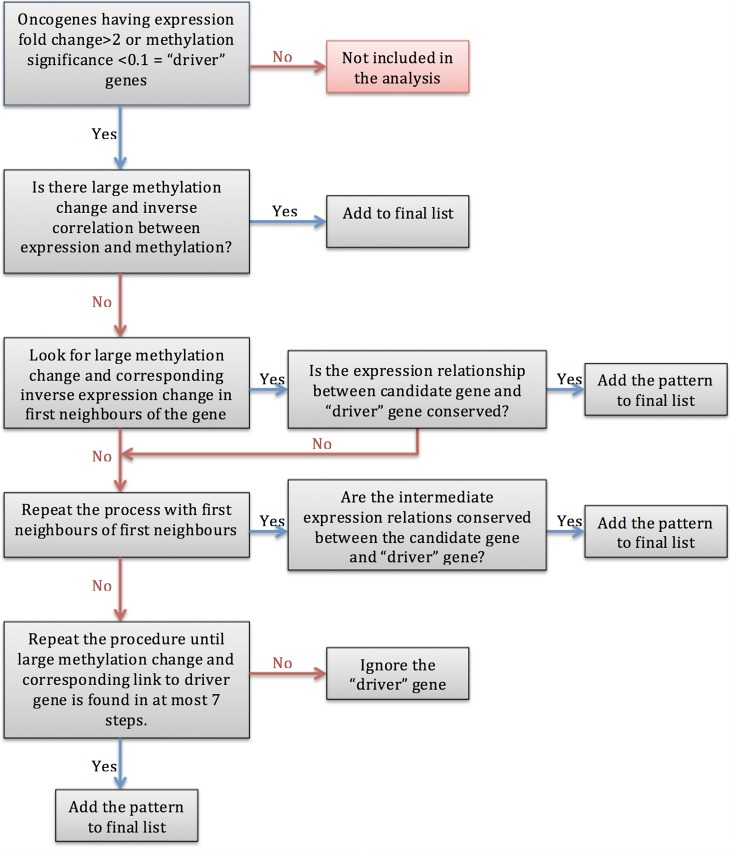
Flow chart of calculating the distance of cancer driver genes from large methylation change. This figure was created for oncogenes, whereas for tumor suppressors we have searched for decrease in expression at the first step instead of an increase. In this procedure, if the fold-change in an oncogene’s expression was >2 or that of a tumor suppressor was <-2, then that gene was added to the short list of cancer driver genes. For each gene on the short list, we searched for a path until we reached a gene showing a change in methylation of >32.2% that caused a corresponding expression fold-change >|2| in inverse order. Moreover, we have also considered activation, inactivation relationships and corresponding expression changes between the genes. If satisfying all these constraints, then that pattern is added to the final list for further analysis.

In this work, we focused on identifying methylation-affected patterns that could potentially result in a cancer state. During this process, we utilized a graph-based, multistep approach, as follows (Fig 1):

- First, only genes with expression fold-changes greater than 2 and methylation FDR < 0.1 were included.
- For each cancer type, we annotated all of the tumor suppressors that were downregulated for that individual cancer type and all of the oncogenes that were upregulated in the same dataset as the “driver genes”.
- In the next step, we calculated all of the paths from each gene to these so-called “driver genes” (either oncogenes or tumor suppressors)
- Most importantly, treating these “driver” genes as starting nodes, we traversed all of the interactions from these genes to their 7^th^ neighbors using breadth-first searches. While investigating each level of neighbor, we searched for inverse correlations between expression and methylation.
- Additionally, we considered the “activation” and “inhibition” relationships between interacting genes; for example, if there was an “activation” relationship between two genes, we searched for a direct relationship between the expression levels of the interacting genes, and if an “inhibition” relationship was observed, we searched for an indirect relationship.
- In order to identify the changes in cancer driver genes that are driven by high methylation change, we have set 32.2% methylation change as “large methylation change” threshold.
- In summary, we utilized three constraints to indicate a path as being “methylation driven”:
  ○ a greater than 15% methylation change
  ○ an inverse correlation between expression and methylation
  ○ the “activation” and “inhibition” relationships were preserved for all genes in the pattern
- Importantly, when more than one path was driven by methylation at the same level, that path was also included at our analysis.
- For each driver gene, the methylation-driven paths with the shortest distances were considered, and all paths with longer distances were discarded.
- Previously, Ozer et al. demonstrated that setting a 15% methylation change threshold for methylation analysis considerably improves the outcome of the analysis [49]. Thus, to determine whether methylation was the primary reason underlying the observed change in the expression level, we used a 15% methylation change as baseline change threshold. As this would enable us to compare high and normal methylation changes, we repeated the abovementioned procedure twice using separate methylation threshold levels: 32.2% and 15%.

**Fig 2:**
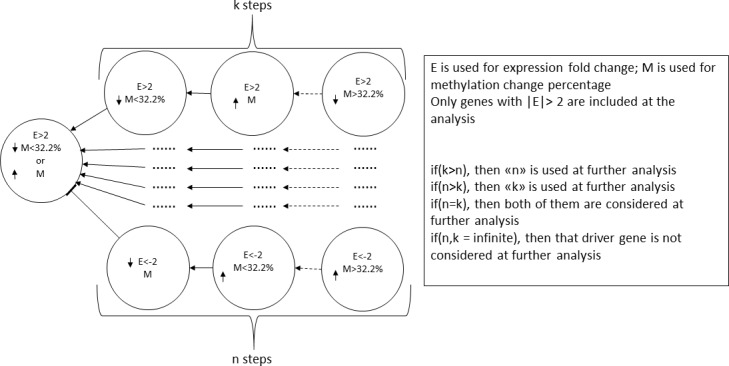
Procedure of calculating the number of methylation-driven interaction steps necessary to reach cancer-related genes. This figure was created for oncogenes, and numerical values can be inversed for tumor suppressors. In order to decide on whether large methylation change is the causative reason behind expression change in driver genes, we have looked for all pairwise relationships from large methylation deregulation to “driver” gene, as all of the intermediate steps between the driver gene and 32.2% methylation change should obey the rules forced by the previous gene. The example scenarios, which pass the defined rules, are shown on this figure. Shortest path from large methylation change to driver gene is only considered at further analysis.

To illustrate our proposed method, an example scenario is provided in Fig 2. Additionally, the number of shared genes among different cancer types was made available for visualization using the Upset R package [50].

## Results

### Differential expression & methylation analysis

We identified the significantly differentially methylated and differentially expressed genes in each cancer type separately. The numbers of differentially methylated and differentially expressed genes are provided in Table 1.

**Table 1:** Numbers of differentially methylated and differentially expressed genes for each cancer type. The rightmost column provides information about the numbers of genes that were both differentially expressed and differentially methylated.

Cancer Type	Number of differentially methylated Genes	Number of differentially expressed genes	Genes with both differential expression and methylation
Cholangiocarcinoma	16,796	9207	8050
Colon adenocarcinoma	15,761	5657	4789
Liver Hepatocellular carcinoma	17,206	5252	4598
Lung squamous cell carcinoma	15,642	8403	6940
Renal papillary cell carcinoma	17,558	5785	5072
Thyroid cancer	14,825	3616	2951

Because we desired to investigate the interplay between methylation and expression, in further analysis, we continued with the genes that showed both significant methylation and expression. More specifically, renal papillary cell carcinoma exhibited the greatest number of differentially methylated genes (FDR<0.1), while the number of differentially expressed genes was highest for cholangiocarcinoma, and the thyroid cancer dataset contained the minimum numbers of genes regarding both differential expression and differential methylation.

We set a methylation change of 32.2% as the large methylation change threshold, and we set a 15% methylation change as the normal methylation change threshold. The latter value was previously defined by Ozer and Sezerman [49]. The numbers of genes showing methylation changes exceeding 15% and 32.2% for each cancer type are illustrated in Table 2.

**Table 2:** Numbers of genes showing methylation changes exceeding 32.2% and 15%. Only the genes exhibiting both differential methylation and differential expression were included in this analysis.

Cancer Type	Number of genes with a large methylation change	Number of genes with a normal methylation change
Cholangiocarcinoma	1792	3128
Colon adenocarcinoma	1503	2270
Liver hepatocellular carcinoma	852	2523
Lung squamous cell carcinoma	1770	3720
Renal papillary cell carcinoma	524	2530
Thyroid cancer	173	828

The results revealed that, comparing the number of genes with large and normal methylation changes, colon adenocarcinoma exhibited the greatest percentage of genes exhibiting a large methylation change (23%), whereas for renal papillary cell carcinoma and thyroid cancer, the percentage of genes with large methylation changes was below 8%. This implies, in colon adenocarcinoma there should be more methylation-linked alterations compared to thyroid cancer or renal papillary cell carcinoma, as the changes in methylome were clearly larger.

### Distances to drivers

Regardless of the direction of the change, it is well established that differences in methylation levels between control and tumor samples exert an important influence on expression levels. However, whether large methylation changes elicit more direct effects and interact more strongly with driver genes remains unknown. To address these issues, we adopted a graph-based approach and calculated the distances between large methylation changes and potentially cancer-causing driver genes by considering pairwise protein-protein interactions. Moreover, we compared the effects of large methylation changes (>32.2%) and normal methylation changes (>15%, <32.2%); the corresponding results are provided in Table 3. In this analysis, we included only the genes that exhibited an inverse correlation between expression and methylation.

**Table 3:** Numbers of genes with large methylation changes (32.2%) and normal methylation change (15%) that reached the driver genes and the average distances between them. Genes with large methylation changes tend to reach to driver genes in higher proportion and in fewer steps compared to the genes with normal methylation change.

Cancer Type	Large methylation change; genes close to driver/genes with path to driver	Large methylation change; Average Distance from driver genes	Normal methylation change; genes close to driver/genes with path to driver	Normal methylation change; average Distance from driver genes	Difference in distance between large and normal methylation change
CHOL	126/1119 = 0.11	2.07	215/3802 = 0.05	2.15	-0.08
COAD	51/796 = 0.06	2.05	51/2978 = 0.02	2.10	-0.05
LIHC	21/571 = 0.04	1.76	58/2854 = 0.02	2.00	-0.24
LUSC	108/923 = 0.12	2.00	179/4559 = 0.04	2.06	-0.06
KIRP	24/371 = 0.06	1.88	65/2754 = 0.02	2.12	-0.24
THCA	12/148 = 0.08	2.58	27/854 = 0.03	2.21	+0.37

The results revealed that the genes with large methylation changes affected driver genes in fewer steps, except for thyroid cancer. Specifically, the average distance from potentially cancer-causing genes was found to be below 2 for liver hepatocellular carcinoma and renal cell papillary carcinoma. When the focus was placed on the proportion of genes that interacted with driver genes in fewer than 4 steps, we observed an overall tendency for more direct interactions when large methylation changes were present. For the genes with normal methylation changes, the proportion of close interactions was 3.00%, while for genes exhibiting large methylation changes the average proportion was 7.83%.

Moreover, with the aim of testing the significance of our findings, we randomly selected differentially altered genes from each cancer type. Subsequently, we examined the average distances from driver genes and the proportions of close interactions with driver genes by applying the same procedure 100 times. During the random selection process, we addressed each cancer type separately, and to determine the number of randomly selected genes, we considered all genes with large methylation changes and a path to a driver of <4, as in our original analysis (numbers of genes for each cancer type: CHOL: 126, COAD: 51, LISC: 21, LUSC: 108, KIRP: 24, and THCA: 12). Consequently, despite repeating the same procedure 100 times with different genes, there was only a single path to one of the drivers for the LUSC and CHOL datasets. More interestingly, for the other four datasets, there were no paths to driver genes. Thus, these results support our findings, and overall, we can state that the genes with large methylation changes interacted with driver genes in higher proportions and in a more direct manner. Thus, large methylation changes may possess more importance regarding cancer etiologies.

### Affected suppressors and oncogenes

To provide insights into cancer mechanisms, we focused on tumor suppressors with decreasing expression levels and oncogenes with increasing expression levels. The tumor suppressors showing expression fold-changes less than -2 and the oncogenes with expression fold-changes greater than 2 and with distances from a driver gene to a large methylation change-driven gene including a maximum of 3 steps are illustrated in Table 4. Only the genes that were shared by at least two cancer types are presented in this table. More detailed results including all of the individual patterns reaching driver genes are provided in S1 Table.

**Table 4:** Numbers of steps between driver genes and genes with large methylation changes for genes that were shared by at least two types of cancer. The genes that were shared and the cancer types sharing these genes can be extracted from this table.

	THCA	CHOL	COAD	KIRP	LUSC	LIVER
**AGTR1**	3	2	2	-	2	1
**IGF1**	-	0	1	1	1	1
**CXCL12**	-	1	1	-	1	2
**FGFR3**	2	0	-	-	0	-
**EPAS1**	-	0	0	-	0	-
**SRC**	-	0	-	0	-	1
**FOXD3**	-	-	0	0	-	0
**PPARG**	1	1	-	-	-	-
**FOXO1**	-	1	-	0	-	-
**CDK4**	-	1	-	-	2	-
**NKX3-1**	-	1	-	-	-	1
**PIK3R1**	-	0	-	-	0	-
**PRKCB**	-	-	1	-	1	-
**EDNRB**	-	-	0	-	0	-

**Fig 3:**
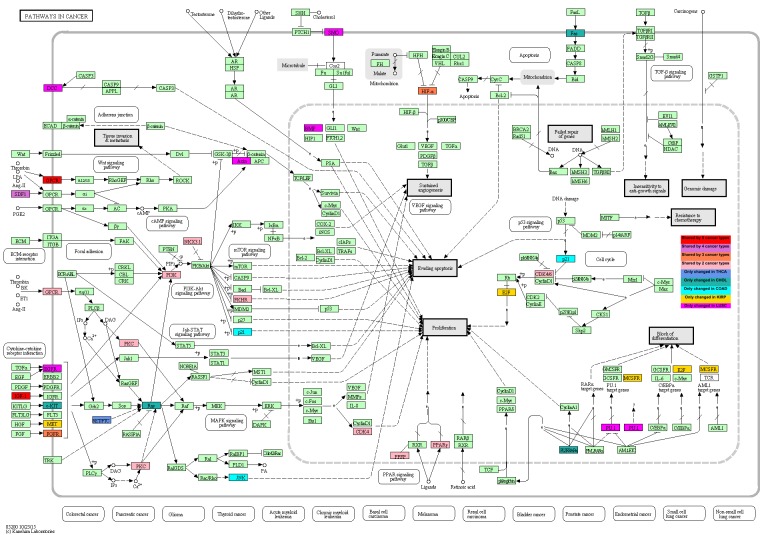
A general picture showing affected genes in KEGG: Pathways in Cancer. The genes are color-coded according to the number or cancer types among which they are shared. Red indicates sharing by 5 cancer types; orchid indicates sharing by 4 cancer types; coral indicates sharing by 3 cancer types; and light pink indicates sharing by 2 cancer types. In contrast, the genes that were affected only in a single cancer type are represented with the following colors: only THCA, cornflower blue; only CHOL, light sea green; only COAD, cyan; only KIRP, gold; and only LUSC, magenta. Unfortunately, there were no genes that were specific to LIHC.

**Fig 4:**
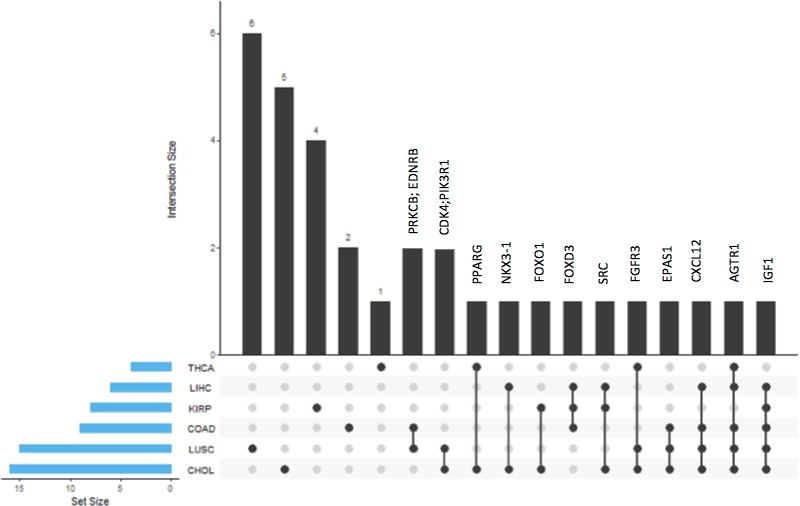
Diagram illustrating the genes shared by different cancers and the cancers among which they are shared. Diagram showing the overlaps of affected oncogenes/tumor suppressors between different cancer types. Black bars are representing the number of genes that are overlapping between different cancers and that are unique to a specific cancer. Gene names for the genes observed in more than single cancer is also shown. Blue bars indicate the total amount of affected oncogenes/tumor suppressors in that specific type of cancer. AGTR1 and IGF1 are observed as affected in 5 different cancer types, improving their significance compared to other oncogenes/tumor suppressors.

Moreover, the identities of methylation-driven genes, the pathways they affected, and the common mechanisms among different cancer types are illustrated according to color in a general illustration of the KEGG: Pathways in Cancer (Fig 3). Additionally, the genes that were shared, the cancer types among which they were shared, and cancer-specific genes are illustrated in Fig 4. Thus, we observed that the AGTR1 (GPCR protein) and IGF1 genes were shared by 5 different cancer types, which are shown in red. CXCL12 was identified in the CHOL, COAD, LUSC and LIHC datasets and is shown in orchid. The genes that were shared by three different cancers (i.e., FGFR3, EPAS1, SRC, and FOXD3) are shown in coral, and the genes that were shared by two types of cancers (i.e., PPARG, FOXO1, CDK4, NKX3-1, PIK3R1, PRKCB and EDNRB) are shown in light pink.

Focusing on the affected genes that were observed only in a single cancer type revealed that the RET gene was affected by high methylation in thyroid cancer (shown in cornflower blue). More specifically, the RET gene interacts with the Ras gene, which is involved in various cancer mechanisms. Examining cholangiocarcinoma, we observed that the HRAS, KIT, ZBTB16, FAS and NCOA4 genes were altered by high methylation (shown in light sea green). Similar to thyroid cancer, the Ras gene was also found to be affected in cholangiocarcinoma. The ZBTB16 gene is active in the blocking of differentiation; thus, methylation-driven abnormalities in ZBTB16 may lead to cholangiocarcinoma. We observed that MAPK10 and CDKN1A were only altered in colon cancer (shown in cyan). Similar to cholangiocarcinoma, four genes that were detected only in renal cell carcinoma play crucial roles in the blocking of differentiation and proliferation (shown in gold). In lung squamous cell carcinoma, we detected 15 genes that were altered by high methylation, and six of these genes were observed to be affected only in this type of cancer (shown in magenta). In contrast, in the hepatocellular carcinoma dataset, we did not detect any gene that was only affected in liver cancer.

Moreover, we conducted copy number analyses of the driver genes that we detected. Because increases in copy number are associated with increases in expression, we asked whether the main reason for the observed expression changes was copy number aberrations, rather than large methylation changes. Copy number analyses corresponding to each driver gene are provided in S2 Table. We focused on the genes showing copy number alterations in more than half of the datasets. Only in the lung cancer dataset were we able to detect such genes. More specifically, the tumor suppressor AGTR1 was found to be increased in 5 of the 7 tumor samples, while the tumor suppressor SPI1 was found to be increased in 4 samples. In contrast, there were 3 samples showing decreased copy numbers of the EPAS1 gene; hence, decreases in the expression of this gene may be related to decreases in copy numbers. Additionally, in the thyroid cancer dataset, copy number analysis of the RET gene revealed decreases in 5 of the 46 samples, while no sample exhibited an increase in the RET copy number. These findings imply that the expression of this gene should be downregulated. However, using our graph-based approach, we were able to associate an increase in RET gene expression with a large decrease in methylation in the proximity of the RET gene.

## Discussion

Our aim in the present study was to identify methylation-driven mechanisms in cancer by adopting a network-based approach. Moreover, we searched for similarities and differences between different cancer types by focusing on the mechanisms affecting cancer-related genes.

In examining the effects of methylation on cancer, driver genes that are crucial for cancer progression should be defined prior to the analysis. Although a variety of driver prediction algorithms are available in the literature, most are based on predicting the effects of mutations, and there is no consensus regarding methylation-based drivers. At this point, the use and annotation of cancer-associated genes depending on their suppressor or oncogene status may provide deeper insight into disease etiology compared with mutation-based prediction algorithms. For this purpose, we extracted a set of genes that had previously been associated with cancer from the KEGG: Pathways in Cancer dataset and through literature mining.

Second, we examined data regarding the differential expression and methylation of these cancer-related genes in six different cancer datasets. Prior to further analysis, we integrated SNP information into the analysis and excluded CpG regions showing mutation frequencies greater than 0.1 in the 1000 Genomes database. During the extraction of differentially methylated and differentially expressed genes, setting an FDR threshold for methylation and fold-change threshold for expression did not decrease the number of short listed genes as desired; therefore, a further decrease in the number of genes was necessary (Table 1). Given this information, setting a methylation change threshold appeared to be promising means of obtaining a clearer picture of the genes that are altered in cancer. Additionally, based on the idea that large methylation changes might exhibit more rapid effects on the mechanisms leading to cancer states, we defined a large methylation change threshold. To this end, we calculated the central value and scaling parameter by pooling all of the data. Consequently, we arrived at a central value of 16.8% and a scaling parameter of 7.77%. Similar to the normal distribution, we set the central value plus two scaling parameters (i.e., a 32.2% methylation change) as the high methylation threshold.

Subsequently, we continued our analysis of the effects of methylation-driven changes on cancer-related genes (driver genes). In this analysis, when no methylation-driven changes in gene expression were identified in the driver genes themselves, we investigated whether integrating protein-protein interaction information could provide additional information about the underlying trigger mechanisms leading to significant expression changes in cancer-related genes. Applying this approach, we used the widely accepted STRING database and calculated the numbers of steps required for genes with methylation changes greater than 32.2% to reach driver genes. To compare large and normal methylation changes, we applied the same procedure utilizing a 15% methylation change threshold. We found that large methylation changes exerted a major influence on the expression of driver genes in fewer steps (1.95 vs. 2.09, except for thyroid cancer) and at a higher proportion (7.83% vs. 3.00%), although these results varied among different cancer types. Moreover, to test the significance of our findings, we applied the same procedure following the random selection of genes 100 times. As a result of this analysis, we found that the ratio of selected genes that reached driver genes in fewer than 3 steps was almost 0 for all of the examined cancer types.

To validate our findings regarding the identified tumor suppressors and oncogenes, we searched for support in the literature. Using our novel graph-based approach, we observed a decrease in the expression level of the tumor suppressor insulin-like growth factor 1 (IGF1), and this decrease was primarily driven by a large methylation change in 5 different types of cancer. Regarding the other cancer types without methylation-driven mechanisms related to IGF1, the expression level of IGF1 was identified as -0.87. Because 0.87 was below our expression status threshold (i.e., a fold-change > 2), this gene was excluded from our analysis in the beginning. When we examined the literature, we found that IGF1 has previously been associated with proliferation and apoptosis, and it has also been demonstrated to play a crucial role in cholangiocarcinoma [51], colon cancer [52], kidney cancer [53], lung squamous cell carcinoma [54] and liver hepatocellular carcinoma [55]. Similarly, angiotensin II receptor 1 (AGTR1) was identified by our method as being shared by 5 different cancer types. AGTR1 is primarily involved in the renin-angiotensin system and has previously been validated as an important tumor suppressor in lung cancer [56], cholangiocarcinoma [57], colon cancer [58] and liver cancer [59]. In the present study, AGTR1 was found to exhibit a decreased expression level; hence, in a maximum of three steps, we identified genes that were altered by large methylation changes that signaled decreases in the expression level of AGTR1. The only cancer that lacked a methylation-driven pattern for AGTR1 was kidney cancer, although the expression change of AGTR1 in kidney cancer was identified as -3.8 and was probably influenced by tissue-specific alterations. In summary, our findings suggest that both AGTR1 and IGF1 are frequently observed as changed in variety of cancers and experimentally validating the hits that we find with our method linking large methylation alteration to these changes may reveal their potentially crucial role on overall cancer progression.

Additionally, it has been previously demonstrated that CXCL12 expression suppresses pancreatic cancer growth and metastasis [60]. In our analysis, the expression of the potential tumor suppressor chemokine ligand 12 (CXCL12) was found to be decreased, possibly due to high methylation, in four different cancer types. In the other two cancer types (particularly thyroid cancer) the CXCL12 gene was not included in the analysis because the change in the expression level of CXCL12 was slightly below our threshold (-0.0996). Regarding the kidney cancer data, the expression of CXCL12 was found to be significantly decreased (-1.15); however the underlying cause of this alteration was not high methylation, but rather, normal methylation (0.18). Moreover, increased methylation-driven expression of the important oncogene SRC was identified in cholangiocarcinoma, hepatocellular carcinoma and colon adenocarcinoma, and SRC has previously been demonstrated to be active in cancer progression [61].

Among the individual, cancer-specific genes that were detected in our graph-based analysis, the well-known RET oncogene has been associated with thyroid cancer progression in numerous studies [62, 63]. Regarding cholangiocarcinoma, HRAS, KIT and FAS have previously been associated with cholangiocarcinoma in the literature [64–66]. Additionally, the most recent efforts directed toward the CDKN1A gene have demonstrated that downregulation of this gene leads to colon cancer progression [67], and similar results have been reported for MAPK10 [68]. Both of these genes were found to be differentially altered by high methylation in colon cancer in our analysis. The C-met oncogene encodes the hepatocyte growth factor receptor [69], and we observed increased MET levels in the kidney cancer data. Additionally, WT1 interacts with the p53 gene, and over-expression of WT1 is associated with renal cell carcinoma [70]. The CSF1R gene has previously been studied and linked to renal cell carcinoma as a potential therapeutic target in patients [71]. Similarly, the E2F1 gene has been suggested as a potential therapeutic target and highlighted as playing a key role in renal cell carcinoma [72]. Although a total of six genes were found to be differentially altered in our liver cancer data, none of these genes were found to be liver cancer-specific in our analysis. More specifically, 5 of the 6 genes were found to be shared with cholangiocarcinoma, which implies that these two cancers share similar mechanisms of cancer progression. In contrast, we identified six cancer-specific genes for lung squamous cell carcinoma, and we have found support in the literature for each of these genes. More specifically, downregulation of the tumor suppressor BMP4 has been linked to LUSC; thus, our method also explained the underlying methylation-driven mechanism [73]. Additionally, changes in EGFR have been associated with lung cancer, and this gene has been recommended as a drug target [74]. Alterations of AXIN2 have been demonstrated to contribute to carcinogenesis, specifically in lung cancer [75], and the downregulation of this suppressor via a large methylation change was successfully detected by our method.

Moreover, we investigated whether increases and decreases in expression were caused by copy number aberrations, rather than large methylation changes. We focused on the driver genes that were identified as crucial to cancer etiology via our graph-based strategy. Because copy number changes in single samples are generally neglected due to corresponding statistical weakness, we focused on genes showing copy number changes in more than half of the datasets. Most notably, we observed genes that met the aforementioned conditions only in lung cancer. More specifically, the AGTR1 and SPI1 genes were identified as showing copy number increases in 5 and 4 samples, respectively, whereas the expression of these tumor suppressors was downregulated in the LUSC dataset. Furthermore, we associated these changes with large methylation increases. Because an increase in copy number is generally associated with greater expression, our findings suggest that large methylation changes were the predominant factors underlying the downregulation of these tumor suppressors, which was supported by copy number analyses. Remarkably, a similar situation was observed for the RET gene in the thyroid cancer dataset; the expression of the RET oncogene was upregulated, whereas 5 tumor samples exhibited decreased copy numbers of RET. Using our approach, we were able to associate the increase in RET oncogene expression with a large methylation decrease, contributing to an improved understanding of the etiology of thyroid cancer.

In contrast to previously applied methods, such as MethylMix and LRpath, our approach integrates protein-protein interaction information in the identification of methylation-driven genes. Additionally, compared to the work of Jiao et al. [76], which also integrates protein-protein interaction information, we have used only matched, paired samples, increasing the confidence of our analysis. In our work, we have focused merely on methylation-driven changes on regulation patterns instead of epigenetically deregulated hotspots. However, we were not able to compare our method with theirs, as there were not enough matched samples for endometrial cancer in TCGA. Recently, many studies have benefited from the use of DNA methylation changes as prognostic markers of disease progression [77–81]. Examples include studies of B cell lymphoma, acute myeloid leukemia, glioblastoma and epithelial squamous cell carcinoma. Basically, Cox regression models that benefit from a set of marker genes were applied in these studies to predict survival and cancer stage. A similar approach can be applied to the genes we have identified, and this method of predicting survival and disease stage can be extended to hepatocellular carcinoma, lung squamous cell carcinoma, colon adenocarcinoma, cholangiocarcinoma and thyroid cancer.

Overall, the synopsis of our findings suggests that in a list of cancer-related genes, methylation-driven pathways either affect the gene itself in a manner that promotes cancer development, or the contributions of the genes to cancer can be explained by cascades of methylation-driven events involving small numbers of interactions that trigger corresponding changes in cancer-related genes. The genes involved in different types of cancer vary, but the manner in which methylation-driven mechanisms affect driver genes exhibits similarities across all cancer types. Our graph-based, integrative approach for identifying methylation-driven patterns provides valuable information regarding cancer etiology, and the genes that are highlighted by our method (especially the oncogenes) may be used as potential therapeutic targets. Although our method reveals important and previously unexplored information about possible methylation-driven changes in different cancer types, whether a large methylation change effectively leads to predicted change in corresponding driver gene in vitro and in vivo remains unknown. Hence, further validation of our important hits may lead to breakthrough findings which can later be used in the field of cancer therapy.

## Supporting information

S1 Table All individual methylation driven patterns reaching to cancer-related driver gene. Table showing affected driver genes, expression changes, methylation changes, methylation positions and number of patterns reaching to that affected driver gene. In addition, all individual patterns are stated with gene names and then with the relationships between them, separated by “_”. First the gene names
(XLSX)

S2 Table Table showing number of samples with decreasing and increasing copy numbers of driver genes. Only the genes which were detected as being affected by high methylation change in maximum proximity of 3 are included at the analysis.
(DOCX)
