# Supplementary material for: An integrative study on the impact of highly differentially methylated genes on expression and cancer etiology
Source: PLoS One. 2017 Feb 8;12(2):e0171694. doi: 10.1371/journal.pone.0171694 (PMC5298317; doi:10.1371/journal.pone.0171694)
Supplement: S2 Table — Only the genes which were detected as being affected by high methylation change in maximum proximity of 3 are included at the analysis. (DOCX) [file pone.0171694.s002.docx]

**Supplemental Table 2)**

| Gene Name | Number of Samples with Increased CN | Number of Samples with Decreased CN | Gene Information |  |
| --- | --- | --- | --- | --- |
| AGTR1 | 0 | 1 | suppressor | THCA - 46 tumour samples |
| FGFR3 | 1 | 6 | oncogene | THCA - 46 tumour samples |
| PPARG | 0 | 4 | suppressor | THCA - 46 tumour samples |
| RET | 0 | 4 | oncogene | THCA - 46 tumour samples |
| FOXO1 | 0 | 0 | suppressor | CHOL - 9 tumour samples |
| PPARG | 0 | 0 | suppressor | CHOL - 9 tumour samples |
| CXCL12 | 0 | 0 | suppressor | CHOL - 9 tumour samples |
| HRAS | 0 | 2 | oncogene | CHOL - 9 tumour samples |
| KIT | 0 | 1 | oncogene | CHOL - 9 tumour samples |
| AGTR1 | 0 | 0 | suppressor | CHOL - 9 tumour samples |
| CDK4 | 0 | 0 | oncogene | CHOL - 9 tumour samples |
| ZBTB16 | 0 | 0 | suppressor | CHOL - 9 tumour samples |
| FAS | 0 | 0 | suppressor | CHOL - 9 tumour samples |
| NKX3-1 | 0 | 0 | suppressor | CHOL - 9 tumour samples |
| EPAS1 | 0 | 1 | suppressor | CHOL - 9 tumour samples |
| FGFR3 | 0 | 2 | oncogene | CHOL - 9 tumour samples |
| IGF1 | 0 | 0 | suppressor | CHOL - 9 tumour samples |
| NCOA4 | 0 | 1 | suppressor | CHOL - 9 tumour samples |
| PIK3R1 | 0 | 1 | suppressor | CHOL - 9 tumour samples |
| SRC | 0 | 0 | oncogene | CHOL - 9 tumour samples |
| PRKCB | 0 | 0 | suppressor | COAD- 15 tumour samples |
| CXCL12 | 0 | 1 | suppressor | COAD- 15 tumour samples |
| IGF1 | 0 | 0 | suppressor | COAD- 15 tumour samples |
| AGTR1 | 0 | 0 | suppressor | COAD- 15 tumour samples |
| MAPK10 | 0 | 0 | suppressor | COAD- 15 tumour samples |
| CDKN1A | 0 | 1 | suppressor | COAD- 15 tumour samples |
| EDNRB | 0 | 0 | suppressor | COAD- 15 tumour samples |
| EPAS1 | 0 | 2 | suppressor | COAD- 15 tumour samples |
| FOXD3 | 1 | 2 | suppressor | COAD- 15 tumour samples |
| FOXD3 | 0 | 0 | suppressor | KIRP - 23 tumour samples |
| FOXO1 | 0 | 0 | suppressor | KIRP - 23 tumour samples |
| MET | 0 | 2 | oncogene | KIRP - 23 tumour samples |
| SRC | 0 | 3 | oncogene | KIRP - 23 tumour samples |
| WT1 | 0 | 1 | suppressor | KIRP - 23 tumour samples |
| CSF1R | 0 | 0 | oncogene | KIRP - 23 tumour samples |
| IGF1 | 0 | 0 | suppressor | KIRP - 23 tumour samples |
| E2F1 | 0 | 3 | suppressor | KIRP - 23 tumour samples |
| BMP4 | 1 | 2 | suppressor | LUSC - 7 tumuor samples |
| DCC | 0 | 0 | suppressor | LUSC - 7 tumuor samples |
| EDNRB | 0 | 1 | suppressor | LUSC - 7 tumuor samples |
| EPAS1 | 0 | 3 | suppressor | LUSC - 7 tumuor samples |
| FGFR3 | 1 | 0 | oncogene | LUSC - 7 tumuor samples |
| PIK3R1 | 1 | 2 | suppressor | LUSC - 7 tumuor samples |
| SMO | 1 | 0 | oncogene | LUSC - 7 tumuor samples |
| EGFR | 3 | 1 | oncogene | LUSC - 7 tumuor samples |
| AGTR1 | 5 | 0 | suppressor | LUSC - 7 tumuor samples |
| PRKCB | 0 | 0 | suppressor | LUSC - 7 tumuor samples |
| CXCL12 | 0 | 1 | suppressor | LUSC - 7 tumuor samples |
| IGF1 | 1 | 1 | suppressor | LUSC - 7 tumuor samples |
| AXIN2 | 0 | 0 | suppressor | LUSC - 7 tumuor samples |
| SPI1 | 4 | 0 | suppressor | LUSC - 7 tumuor samples |
| CDK4 | 1 | 1 | oncogene | LUSC - 7 tumuor samples |
| FOXD3 | 4 | 2 | suppressor | LIHC - 39 tumour samples |
| IGF1 | 0 | 2 | suppressor | LIHC - 39 tumour samples |
| SRC | 1 | 3 | oncogene | LIHC - 39 tumour samples |
| CXCL12 | 2 | 3 | suppressor | LIHC - 39 tumour samples |
| NKX3-1 | 1 | 3 | suppressor | LIHC - 39 tumour samples |
| AGTR1 | 1 | 1 | suppressor | LIHC - 39 tumour samples |
